# Regeneration of Pancreatic *β*-Islet Cells in a Type-II Diabetic

**DOI:** 10.1155/2018/6147349

**Published:** 2018-09-05

**Authors:** Edwin C. Jones, J. Craig Rylands, Cortney L. Jardet

**Affiliations:** ^1^Department of Veterans Affairs, Knoxville Outpatient Clinic, 8033 Ray Mears Blvd., Knoxville, TN 37919, USA; ^2^Summit Medical Group, 7211 Wellington Dr. #201, Knoxville, TN 37919, USA; ^3^Endocrinology Consultants of East Tennessee, 1450 Dowell Springs Blvd., Suite 300, Knoxville, TN 37909, USA

## Abstract

A case report is presented in which a type-II diabetic patient significantly improved his dysfunctional *β*-islet cells using a combination of a strenuous exercise program, cyclical ketogenic diet, and oral GABA/probiotic supplementation. The patient was diagnosed with type-II diabetes at the age of 41 which then progressed through a typical series of treatment changes over 14 years. Treatment periods consisted of metformin therapy alone for 4 years followed by a metformin/glyburide combination therapy for 6 years, and eventually an insulin/metformin combination therapy for 4 years. One year after the initiation of insulin, the patient increased the level of strenuous physical activity (hiking and weight lifting) and adopted a ketogenic diet. Oral GABA and probiotic supplementation were also initiated at the age of 52.7. By the age of 55, the patient no longer required any insulin and is currently being managed with metformin alone. C-peptide values indicate a functional improvement of the *β*-islet cells during the time of insulin/GABA/probiotic treatment.

## 1. Introduction

Diabetes mellitus is a disease diagnosed in millions of individuals worldwide. The adult onset form of this disease, i.e., Type II, is caused by insulin resistance. Over the course of this disease many will go on to develop exogenous insulin dependence. Once insulin dependent, it is rare for a patient to decrease their need for exogenous insulin. The exceptions are almost entirely limited to pregnancy in patients with pregestational diabetes mellitus [1]. In these patients decreasing insulin requirements can indicate placental dysfunction [1]. This report discusses an adult male who needed up to 60 units of long-acting glargine insulin daily for four years and whose insulin requirement was completely resolved.

## 2. Case Report

**Figure 1:**
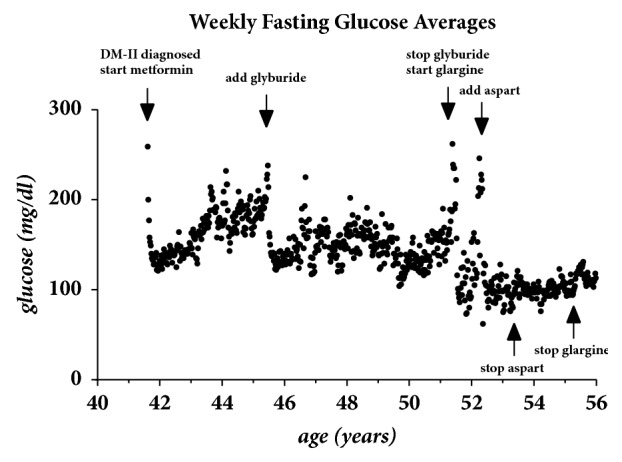
Weekly fasting glucose averages over a 14-year period with major treatment changes being indicated by the arrows.

This case discusses the effect of strenuous exercise in improving the glycemic control of a type-II diabetic. A Caucasian male patient who developed diabetes at the age of 41 initially controlled with oral agents and progressing to insulin by the age of 51 has to a great extent reversed this condition with strenuous physical exercise and diet. This patient previously underwent genetic testing at 23andme.com and was identified as being genotype TT on the gene TCFL2 (SNP rs7903146 of Chromosome 10q). This genotype is a 92% predictor of DM-II. Genotype TT predicts a decreased insulin secretion and increased hepatic glucose production [2, 3].  Figure 1 illustrates the progression of his diabetes over a 14-year period. This graph is a plot of fasting glucoses averaged over one week against the age of the patient. The points where oral agents metformin and glyburide were started and stopped are indicated by the arrows. The points where the long-acting insulin glargine and the short-acting insulin aspart were started and stopped are similarly shown. Since stopping both insulin types by age 55.3 years the patient's fasting glucose values have only slightly rebounded to the average seen shortly after starting the metformin at the age of 41.

At the time of original diagnosis and prior to treatment at age 41, the patient weighed 78 kg and had a BMI of 24.0. C-peptide and antibody tests were made to confirm the specific type of diabetes. The c-peptide was determined to be 3.1. The antibody tests anti-GAD, insulin autoantibodies, and islet cell IGG autoantibodies were all found to be negative, i.e., <1.0 U/ml. At the age of 46, the GAD-65 autoantibody test was repeated and remained negative. It is worth pointing out that the patient received a full physical at the age of 40 and a CMP indicated no abnormal glucose values indicating the diabetes was diagnosed relatively quickly.

**Figure 2:**
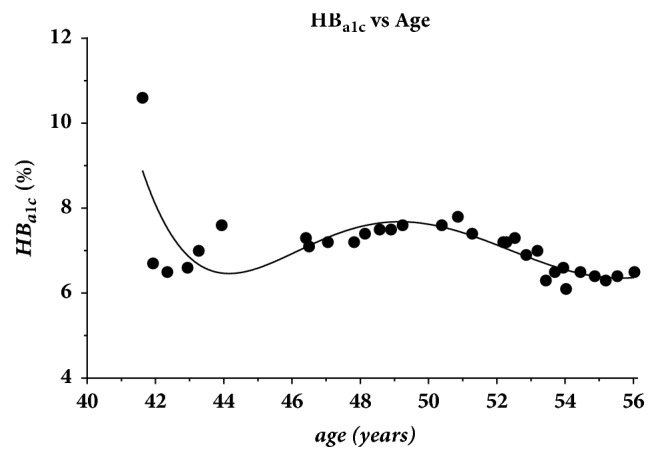
HB_a1c_ values as a function of age.

The patient progressed through a series of treatment changes over the next 14 years. Metformin was initiated at the age of 41 following the initial diagnosis of DM-II. By the age of 45, the sulfonylurea glyburide was added. Over the next 6 years, the efficacy of glyburide gradually waned with his HB_a1c_ peaking at 7.9 by the age of 50 years in Figure 2. Glyburide was changed to long-acting glargine insulin at the age of 51 years. At the age of 52, short-acting aspart insulin was added for improved glycemic control which was needed for a period of one year. Combined glargine and aspart doses exceeded 100 units daily for one full year. Finally, between the ages of 53 and 55 years, the patient was maintained on long-acting glargine and metformin. The patient began recording daily carbohydrate intakes at the age of 52 when the short-acting aspart was initially added. These recorded carbohydrates are continuing to be recorded to date and are composed of the total carbohydrate contents minus one-half of the fiber intakes. Three months following the discontinuation of all insulin at age 55.3 years, the patient's HB_a1c_ rebounded by 0.3% to 6.6% as shown by the slight increase in the final two points in Figure 2. This remains within the range of management with other oral, noninsulin, glucose lowering medications not available at the time of the initial diagnosis.

**Figure 3:**
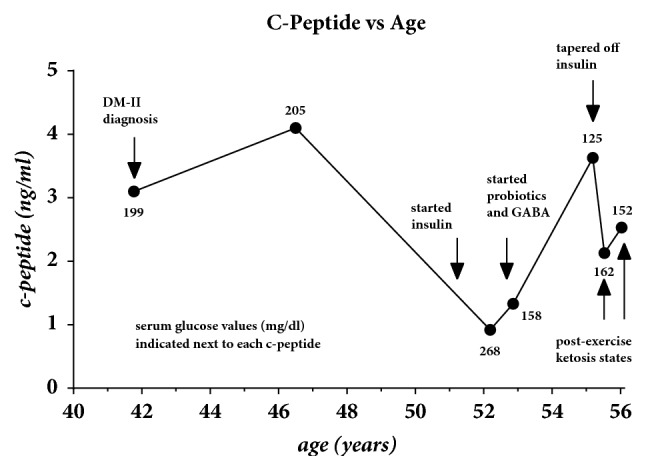
C-peptide values versus age. Arrows indicate the time of DM-II diagnosis, initiation-discontinuation of insulin, and the introduction of probiotics/GABA supplementation. The final two arrows indicate c-peptides during a postexercise ketosis state when insulin need is presumably lower. The numbers adjacent to each of the c-peptide points are the serum glucose values in mg/dL at the time of these tests.

After 4 years of insulin therapy and increase in strenuous exercise, the patient began to notice a gradual and progressive decrease in the need for long-acting glargine insulin. By the age of 55.3, the patient was taken off all insulin types. A c-peptide test was repeated showing an increase from 0.92 (age 52.2) to 3.63 (age of 55.2) as shown in Figure 3. This suggests a significant functional improvement of the *β*-islet cells. No case reports with such significant improvements in the c-peptide in humans have been found in the literature. The figure shows that the latest c-peptide is actually higher than the first c-peptide of 3.1 measured at the time of initial diagnosis of diabetes. Two additional c-peptide follow-up tests were repeated at ages 55.53 years and 56.03 years with resulting values of 2.13 ng/ml and 2.53 ng/ml, respectively. Although these are decreased below the peak at age 55.2, both of these tests were on days following strenuous 27.7 km hikes with the patient being in a postexercise ketosis state. These were confirmed with urine ketones testing in the range of 15-40 mg/dL. None of the other c-peptide tests of record were made on dates where the patient was in a postexercise state and presumably in a lower insulin requirement state. Although a biopsy with histology of the islet cells could provide insight into the detailed mechanism for the functional improvement of the *β*-islet cells, this procedure carries too many risks for a nonterminal condition such as diabetes mellitus. Therefore, the standard test for assessing insulin production is the c-peptide.

**Figure 4:**
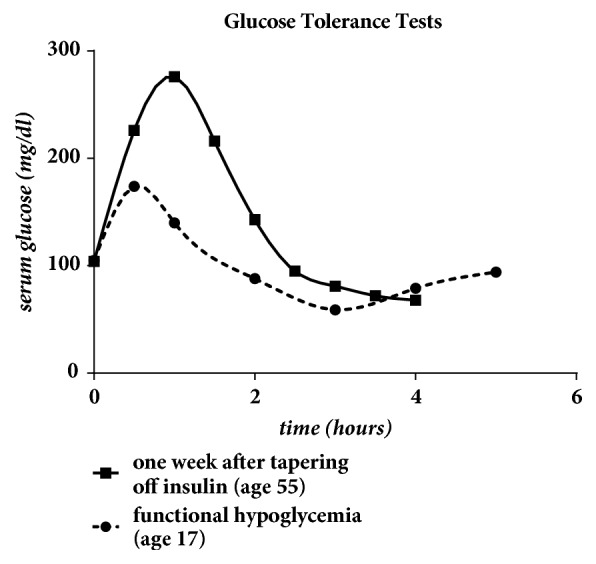
Glucose tolerance tests at the ages of 17 and 55 following the ingestion of 50 grams of dextrose. The patient was taking no medications at age 17 and metformin at age 55.

A glucose tolerance test following the administration of 50 grams of dextrose (d-glucose) was conducted one week after the patient was completely off all exogenous insulin. The test indicated a peak in glucose at one hour following the dextrose administration with serum glucose values dropping below 100mg/dl after 2.5 hours. This confirmed the presence of functional *β*-islet cells. In Figure 4, this test was compared to a glucose tolerance test conducted when the patient was 17 years old. At that age, the patient reported experiencing mild functional hypoglycemia following the ingestion of a large carbohydrate meal.

A review of all medications, supplements, diet, and physical activities was conducted to help identify the factors leading to the functional improvement of the patient's *β*-islet cells. The patient reported a lifelong hobby of long distance hiking and intermittent weight lifting. The latter was reported to be employed to help offset the decrease in upper body muscle mass following frequent long distance hiking.

### 2.1. Medications

Medications included metformin therapy for 4 years followed by metformin/glyburide combination therapy for 6 years, and insulin/metformin combination therapy for 4 years, followed by metformin alone. Insulin need was eliminated by the age of 55.3 following the functional improvement of the patient's *β*-islet cells. Other medications include quinapril 40mg daily and pravastatin 20mg daily. The patient's average biometrics over the 14-year period include a total cholesterol of 165 mg/dl, LDL 87 mg/dl, BP 110/70 mmHg, and resting pulse of 60 bpm.

### 2.2. Supplements

Over-the-counter supplements include magnesium 400mg daily, fish oil 2g daily, GABA 1.5mg at bedtime, and daily probiotics. Probiotic capsules included at minimum strains of both* Lactobacillus* and* Bifidobacterium*.

### 2.3. Diet

**Figure 5:**
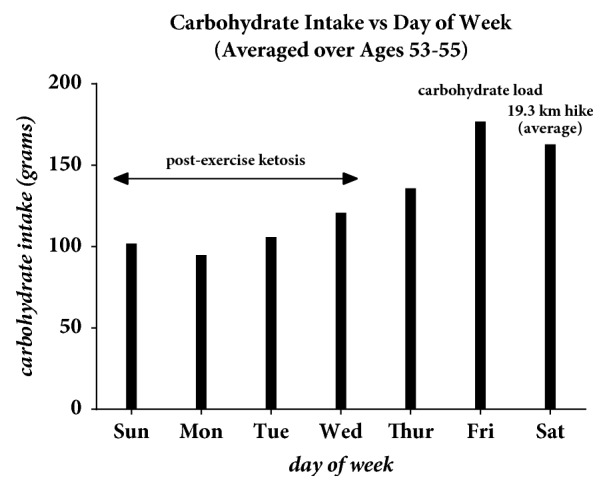
Carbohydrate intake sorted by day of week between the ages of 53 and 55 years.

A low carbohydrate ramping diet used by many weight lifters was adopted at age 53 years.  Figure 5 illustrates the number of carbohydrates sorted by day of week over a two-year average between the ages of 53 and 55 years. On Saturday an average of a 19.3 km hike was made driving the patient into a postexercise ketogenic state lasting into Wednesday or Thursday as confirmed by urine acetoacetic acid ketone test strips. Ketosis was identified by the presence of urine ketones ≥ 5 mg/dl in the absence of urine glucose.

### 2.4. Physical Activities

The patient reported a lifelong history of physical activity of varying degrees. The most common physical activities were reported to be hiking and weight lifting. The patient trained as a scientist, and due to a significant variation in his available spare time for external activities, the amount of hiking varied from a low of 145 km hiking per year to a high of 1223 km hiking per year in mountainous terrain. The patient also maintained detailed hiking logs with totaled annual hiking distances. The patient also reported weight lifting an average of five hours weekly and hiking an average of 19.3 km on the weekends between the ages 53 and 55 (two full years).

**Figure 6:**
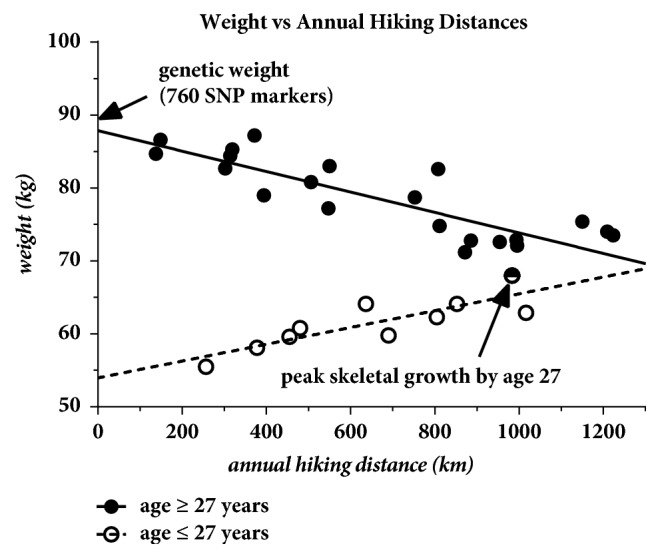
Weight plotted against annual hiking distances in mountainous terrain. The change in slope in these data at age 27 indicates that his maximum skeletal growth was attained by that age.

At age 55.1 following a prediction from 23andme.com based on 760 genetic SNP markers that his predicted genetic weight was 89.8 kg (computed for age 45 years), the patient plotted the actual recorded weights against the annual hiking distances. As shown in Figure 6, these data revealed a very a strong correlation with weight decreasing at a rate of 1 kg for every 71 km of annual hiking in mountainous terrain. The data also indicated that peak skeletal growth was achieved by the age of 27 (marked by arrow) as indicated by the rapid change in slope in the data at the age of 27 years. These data will serve as a baseline for additional studies to follow.

**Figure 7:**
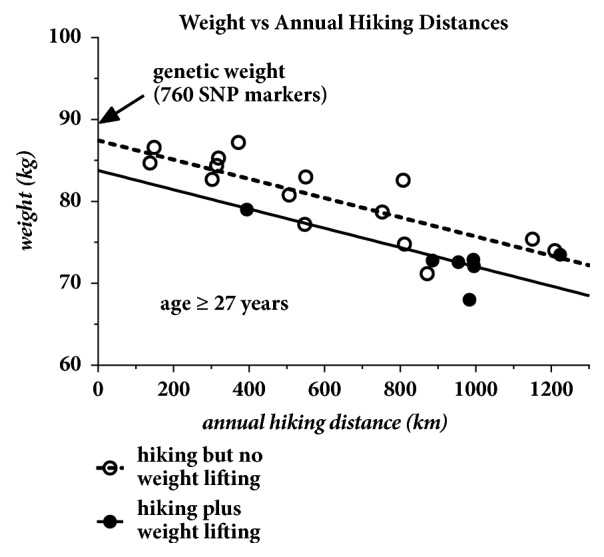
Weight beyond age 27 sorted according to whether or not the patient was actively engaged in a weight lifting program. The data indicate a decrease of 3.6kg in overall weight during the weight lifting years.

An additional trend was also extracted from these annual weight data. For the years beyond age 27, the data shown in Figure 7 were further subdivided into the years the patient was actively engaged in a weight lifting program and compared to those years with no active weight lifting. A typical weight lifting year was reported to be composed of five workouts per week lasting one hour each and these data are shown as solid circles. For comparison, the nonweight lifting years are shown as open circles. These data reveal that the original trend is actually two parallel trends with weight lifting further reducing the patient's overall weight by an additional 3.6 kg.

**Figure 8:**
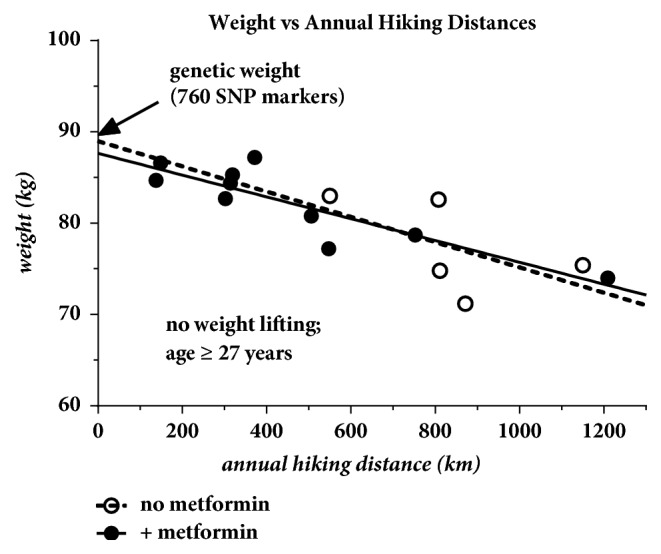
Weight beyond age 27 sorted according to whether or not metformin therapy was prescribed. The lack of significant separation in the curves strongly suggests that this is a weight neutral medication. There are an insignificant amount of data for the weight lifting years to be included at this time.

These weight trends also hold significant potential in evaluating the long term impact of various medications on weight. There were a significant amount of weight data for the impact of metformin on the patient's weight beyond the age of 27 with no weight lifting. These data are plotted in Figure 8. This figure indicates no significant change in the patient's weight with or without metformin therapy supporting that this is a weight neutral medication. Other medications could similarly be assessed in the future as more data are collected.

## 3. Discussion

During the four years of combined insulin and metformin therapy the patient was treated with both long-acting glargine and short-acting aspart. Insulin was started at the age of 51.35 years. Insulin doses needed to optimally control the patient's glucose reached peak doses at the age of 51.50 years for glargine and at the age of 52.40 years for aspart. The highest doses were 60 units in the evening for glargine and 70 units divided over meals for aspart. Following these peaks the dose requirement for both insulin types gradually decreased with the patient being tapered off aspart by the age of 53.25 years and tapered off glargine by the age of 55.30 years. The patient is currently being managed with metformin alone. A followup three months following the discontinuation of insulin at age 55.57 years revealed that the HB_a1c_ increased by just 0.3%, i.e., an increase from 6.3% to 6.6%. The patient's metformin dose was increased from 1000mg daily to 1500mg daily at the time of this three-month followup.

Just prior to being tapered off glargine, a c-peptide was collected at the age of 55.16 years to confirm the presence of native insulin. The result was normal with a value of 3.63 mg/dL. Furthermore, the rebound of c-peptide was found to be 1.092 ng/mLyr as shown by the rise in the c-peptide between ages 52.1 and 55.2 years (Figure 3) indicating a functional improvement of *β*-islet cells. This prompted a medical literature review into the possible mechanisms responsible for the improvement in the patient's *β*-islet cell functioning.

During the latter three years of insulin therapy and postinsulin therapy, the patient reported an increase in hiking distances and a resumption of weight lifting. The patient reported hiking 148 km, 394 km, 885 km, 993 km, and 1223 km at the ages of 51, 52, 53, 54, and 55 years, respectively. Weight lifting was resumed at the age of 52.70 years. This led to dramatic decreases in weight from 85.3 kg to 73.0 kg. The patient also reported restarting the weight lifting program to gain lean body mass to help offset loss of upper body muscle mass due to the long distance hiking. Over-the-counter probiotics and GABA 1.5g daily were started at the age of 52.70. These are frequently used by weight lifters to help encourage the growth of lean body mass [4–6]. Carbohydrate cycling was initiated at the age of 52.70 years which is also used by many body builders to encourage leaner body mass [7]. During days of lower carbohydrate intakes, cheese was included in the diet and cheese is also noted to be a natural source of GABA [8].

A review of the medical literature revealed a reversal of diabetes in several mouse studies. Many studies focused on the effects of GABA on nonneuronal cells such as the pancreatic *β*-islet cells. Within these pancreas islets, GABA is known to decrease glucagon secretion from the *α*-islet cells and increase insulin secretion from the *β*-islet cells [9]. Furthermore, GABA has been shown to stimulate proliferation of the *β*-islet cells as well as providing protection from the deleterious effect of hyperinsulinemia [10–14]. The pancreatic GABA signaling is reported to be altered in type 2 diabetes [15]. Furthermore, gut microbes have recently been shown to produce the neurotransmitters GABA, norepinephrine, dopamine, and serotonin [16]. Species of* Bifidobacterium* and* Lactobacillus* produce high concentrations of GABA [16]. In contrast, less common gut bacteria, e.g.,* Flavonifractor* sp., consume GABA reducing the GABA concentration in the gut [17, 18]. Therefore, probiotics containing species of* Bifidobacterium* and* Lactobacillus* presumably have an indirect influence on the pancreatic islet cell functioning.

More recently Cheng et al. showed that exposing mice to repetitive cycles of a fasting mimicking diet could reverse the *β*-islet cell failure through the expression of the Ngn3 messenger [19]. The Ngn3 messenger is also believed to hold the potential of regenerating the *β*-islet cells in humans [19] assuming that the chromosomal telomeres of these *β*-islet cells have not yet reached a critically short length leading to cell senescence [20, 21]. It is suggested from these research studies that the combinations of GABA, probiotics, and carbohydrate cycling diet during the four years of insulin therapy are responsible for the functional improvement of the patient's *β*-islet cells as indicated in the c-peptide change previously described in Figure 3.

## 4. Conclusion

A patient diagnosed with type-II diabetes at the age of 41 who progressed through a typical series of treatment stages began showing signs of disease reversal over a decade later. Improvements of the c-peptide indicate a functional improvement of pancreatic *β*-islet cells around the time of an increase in strenuous exercise, the initiation of oral probiotics and GABA supplementation, and the initiation of a strict carbohydrate cycling diet. A review of the medical literature indicates that these findings have been seen in several mouse studies but limited information has been reported in human studies. The findings presented in this case report strongly suggest that these treatment techniques should be investigated further. Significant improvements in a disease commonly seen in the population would improve the lives of many patients and greatly reduce the financial burdens imposed by this disease on society.
